# From acute neglect to chronic constructional deficits: parietotemporal contributions to long-term post-stroke impairments

**DOI:** 10.1093/braincomms/fcaf477

**Published:** 2025-12-04

**Authors:** Jie Song, Eugénie Cataldo, Marine Thomasson, Arnaud Saj, Patrik Vuilleumier, Roberta Ronchi, Ilaria Sani

**Affiliations:** Department of Basic Neurosciences, Faculty of Medicine, University of Geneva, 1202 Geneva, Switzerland; Department of Basic Neurosciences, Faculty of Medicine, University of Geneva, 1202 Geneva, Switzerland; Department of Clinical Neurosciences, Hôpitaux Universitaires de Genève, University of Geneva, 1202 Geneva, Switzerland; Department of Basic Neurosciences, Faculty of Medicine, University of Geneva, 1202 Geneva, Switzerland; Departement de Psychology, Faculty of Arts and Sciences, University of Montreal, H2V 2S9 Montreal, Canada; Department of Basic Neurosciences, Faculty of Medicine, University of Geneva, 1202 Geneva, Switzerland; Department of Basic Neurosciences, Faculty of Medicine, University of Geneva, 1202 Geneva, Switzerland; Department of Clinical Neurosciences, Hôpitaux Universitaires de Genève, University of Geneva, 1202 Geneva, Switzerland; Department of Basic Neurosciences, Faculty of Medicine, University of Geneva, 1202 Geneva, Switzerland

**Keywords:** visuospatial processing, unilateral spatial neglect, object processing, lesion mapping, disconnection mapping

## Abstract

Patients with acute hemispheric stroke exhibit various visuospatial impairments. While many recover rapidly, others remain impaired. Better defining which symptoms characterize the acute and chronic phases and which brain areas and connections are implicated could help to improve diagnostic and rehabilitation tools and inform effective rehabilitation strategies. Here, we report a systematic anatomo-functional study of two populations of acute and chronic hemispheric stroke patients (cross-sectional design). Patients were examined by a series of neuropsychological tests assessing different post-stroke clinical manifestations in the visuospatial domain. We first performed a statistical factorial analysis of patients’ behavioural performance across tests to break down symptoms into coherent profiles of co-varying deficits and determine whether any factors may be specific to each post-stroke phase. We then conducted voxel- and atlas-based lesion-symptom mapping, as well as disconnection-symptom mapping in the two populations. We found different patterns of behavioural impairment across groups, with acute symptoms mostly characterized by lateralized attentional deficits and chronic symptoms manifesting as constructional spatial impairments. Lesions to and/or disconnections of frontal and precentral gyri correlated with lateralized visuospatial symptoms in the acute but not chronic phase, whereas lesions to and/or disconnections of temporoparietal areas correlated with constructional deficits in the chronic phase. Our results indicate that constructional spatial deficits and damage/disconnection of dorsoventral higher-order visual areas most pervasively impair stroke patients in the long term. Such deficits might be overlooked or disregarded by rehabilitation strategies focusing on the (mainly acute) lateralized component of their visuospatial deficits and ignoring concomitant, more object-based deficits. This work may help design more specific diagnostic tests and guide future rehabilitation strategies, ultimately promoting better and more extensive recovery beyond lateralized deficits in attention and spatial awareness.

## Teaser

Song *et al.* report that visuospatial impairments after stroke follow distinct anatomo-behavioural patterns across recovery phases. Acute symptoms primarily reflect lateralized attention deficits linked to frontoparietal damage and interhemispheric disconnections; chronic impairments involve constructional deficits linked to temporoparietal damage and disconnections. Findings suggest distinct diagnostics and rehabilitation should target early and persistent deficits.

## Graphical Abstract

**Graphical Abstract:**
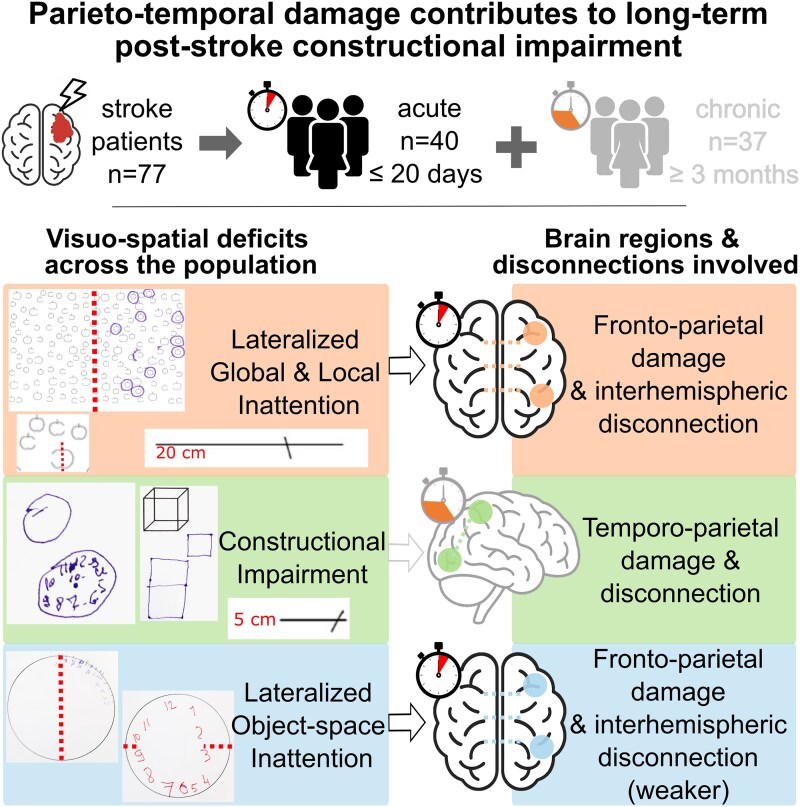

## Introduction

Stroke is one of the leading causes of disability worldwide,^1,2^ placing a significant burden on individuals, caregivers, healthcare systems and society as a whole. As medical advancements improve survival rates, there is a corresponding increase in long-term impairments that affect the quality of life for patients, with differential needs for short-term and long-term interventions.^2-5^

Among the many consequences of stroke, visuospatial impairments are particularly debilitating,^6,7^ impacting patients’ ability to interact with their environment and perform everyday tasks. These impairments can manifest in various forms, ranging from difficulties in spatial awareness and attention to challenges in recognizing and manipulating objects. Such deficits may severely hinder independence and routine activities, yet not all of them have been examined and understood to the same extent.

Unilateral spatial neglect (USN), a condition where patients fail to attend to, perceive and/or represent one side of space, is among the most well-documented visuospatial deficits following a stroke, particularly those in the right hemisphere.^8-10^ This condition has garnered considerable attention in the scientific literature due to its relatively high prevalence and its dramatic impact on patients’ lives.^6^ Extensive literature indicates that symptoms of USN are multivarious and linked to damage in several areas, including the right inferior parietal lobule,^11,12^ superior temporal gyrus,^13^ frontal regions,^9,14^ basal ganglia^15,16^ and frontoparietal disconnection after damage to the superior longitudinal fasciculus.^17,18^ Past studies have provided valuable insights into the neural mechanisms underlying spatial attention and the possible strategies for rehabilitation. However, the presence of USN may also affect the evaluation of other concomitant cognitive disorders, possibly leading to an underappreciation of other significant visuospatial deficits.

During interactive behaviours in space, objects constitute a major element to be perceived and acted upon, and their disturbance deserves consideration. Object-based deficits after stroke manifest in variable forms, ranging from inaccurate integration of objects’ constituents, their physical extent or their internal representation. Within the spectrum of symptoms of USN, object-centred (allocentric) neglect, for example, refers to the inability to attend to one side of an object, regardless of its position relative to the patient’s egocentric space; this typically arises from damage to the right hemisphere^19-21^ within superior temporal regions,^11^ the angular gyrus^13^ and parts of the posterior superior temporal gyrus.^22^ Another object-based disorder is visuoconstructional apraxia, which is characterized by a patient’s inability to assemble or construct objects—whether drawing, building or arranging items—in space; this is primarily associated with damage to the parietal lobe, especially the right side when the capacity of spatial analysis is compromised,^23^ but can also involve the frontal lobe,^24,25^ temporal lobe,^24,26^ supramarginal gyrus^24,26^ and occipitoparietal junction.^24^ These specific object-based deficits are less frequently studied, even though they may impact the patients in their daily life activities. These deficits may often be underdiagnosed in clinical settings^19,27^ and infrequently addressed in rehabilitation efforts,^28^ yet they may persist longer after a stroke.^19,20,29^

A critical aspect of post-stroke recovery is indeed the variability in how patients regain their functions: some individuals recover rapidly while others continue to experience significant impairments long after the initial event; moreover, some deficits improve quickly, but others endure over time. While the recovery pattern of egocentric neglect is relatively well characterized,^27,30-32^ the impact of object-centred visuospatial deficits such as allocentric neglect or constructional disorders in the acute versus chronic phase remains largely unexplored and is a major focus of the current study. Furthermore, the brain structures involved in these potentially persisting deficits remain poorly understood. Understanding the distinction between deficits associated with acute and chronic stroke phases, both in terms of symptoms and brain substrates, is essential for developing effective diagnosis strategies and rehabilitation interventions.

We hypothesize that, beyond egocentric USN, other visuospatial deficits may be a significant component of cognitive impairments after brain damage and could persist longer into the chronic phase. Additionally, we surmise that different brain structures may contribute to distinct visuospatial symptoms at various stages of stroke recovery. To test these hypotheses, in the present study, we systematically investigated the anatomo-functional correlates of visuospatial impairments in stroke patients through (i) a detailed cognitive characterization of deficits, (ii) a direct comparison of acute and chronic phases and (iii) a precise analysis of structural brain changes, including voxel-level mapping, anatomo-functional classification, tract disconnection indices for whole-brain data and area-to-area connections. Specifically, we combined neuropsychological testing with machine learning-based lesion-symptom mapping (LSM) and disconnection-symptom mapping to identify the distinct brain regions and connections associated with different visuospatial deficits at various stages post-stroke. By elucidating the neural underpinnings of these impairments and their progression from acute to chronic phases, our study may also help to inform diagnostic accuracy and rehabilitation strategies, ultimately improving the quality of life for stroke survivors.

## Materials and methods

### Participants

**Figure 1:**
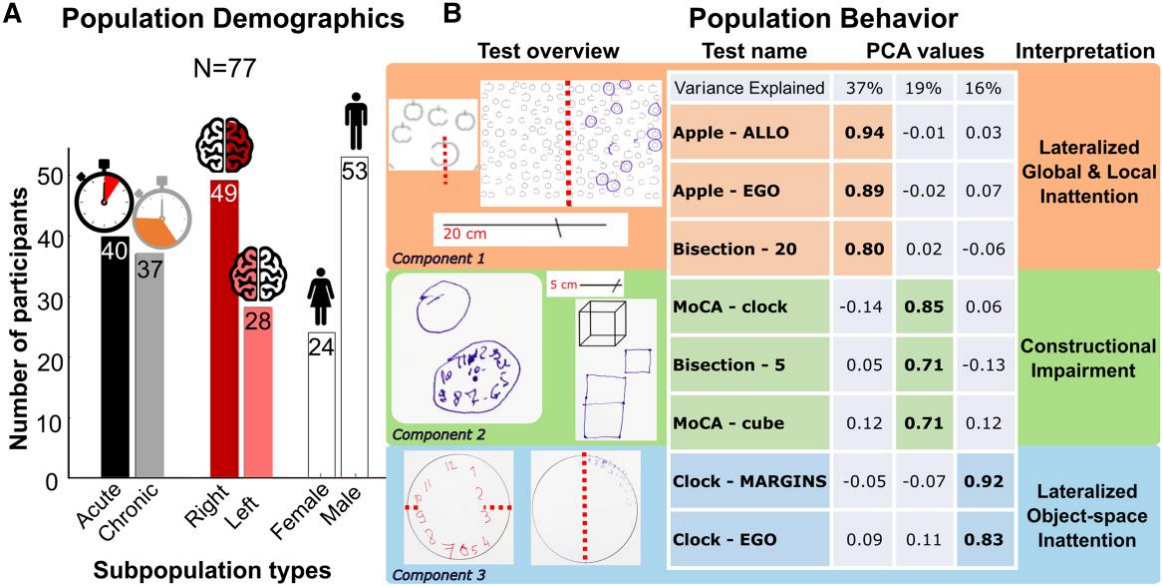
**Population demographics and behaviour.** (**A**) Patients’ number according to time after stroke, lesion side and gender. (**B**) PCA results of behavioural symptoms across the whole population (*N* = 77): the first two columns show individual examples of performance and test name; the middle columns show factor loadings of each test score; the last column shows component interpretation. PCA, principal component analysis; Apple, apple cancellation test; ALLO, allocentric; EGO, egocentric; Bisection-20, line bisection test in 20 cm length; MoCA-clock, Montreal Cognitive Assessment-clock drawing test; Bisection-5, line bisection test in 5 cm length; MoCA-cube, Montreal Cognitive Assessment-cube copying test; Clock, clock drawing test.

We recruited retrospectively 97 first-event unilateral stroke patients from Geneva University Hospital (HUG, Switzerland) for a cross-sectional design study. Twenty patients were excluded due to either general cognitive impairments (3), missing neuropsychological tests (9), difficulties in understanding the tests (3), bilateral brain lesions (2), missing lesion maps (1) or associated cerebellar lesions (2). Thus, 77 patients (53 M/24 F, 35–88 years old, 49 right/28 left hemisphere) with magnetic resonance imaging (MRI) images and visuospatial test scores were included in the present study (Fig. 1A). Forty patients were tested in the early period after stroke (i.e. ≤20 days after stroke; mean duration of disease 4 days; mean age 64.1 ± 14.2 years old with range 29–88; 36 right-handed; 12 females; 24 right hemispheres), while 37 were chronic patients (i.e. ≥3 months after stroke; mean duration of disease 283 days; mean age 60.6 ± 9.9 years old with range 35–77; 26 right-handed; 12 females; 25 right hemispheres). None of them had a history or evidence of previous neurological or psychiatric diseases or global cognitive impairment/decline on a standard neuropsychological exam. Thirteen patients had motor impairment of the upper limb, and all used their unaffected, contralesional limb to perform the tasks. All patients in each group performed all the tests described below. Part of the data have been published somewhere else.^33-36^ Patients provided informed consent following the Declaration of Helsinki and procedures established by the University of Geneva Review Board (CE 12-126, CE 11-250 and CE 2021-01117).

### Neuropsychological assessment

Visuospatial deficits were assessed using a systematic battery of standard paper-and-pencil tests that could be easily administered in a clinical setting. These tests included the line bisection test, the apple cancellation test, the clock drawing test and two scores from the Montreal Cognitive Assessment (MoCA); see Fig. 1B (see also Supplementary Table 1 for a list of tests).

In the ‘line bisection test’, patients were asked to mark the middle of four horizontal lines presented individually on an A4 sheet of paper (two were 5 and two 20 cm long^37,38^). The score was the magnitude of rightwards or leftwards deviation from the true centre (in millimetres). This test assesses a laterality bias at two different spatial scales (i.e. lengths).

In the ‘apple cancellation test’,^39^ patients were asked to mark all drawings of full apples among other apples with an opening on either the left or right side. Two scores were obtained to reflect egocentric neglect (omissions of targets on the contralesional side of the sheet) and allocentric object-based neglect (incorrect selection of apples opened on the contralesional side of the apple—false positive). Egocentric neglect severity was determined as a continuous variable by averaging the *z*-transformed egocentric centre of cancellation (CoC).^40^ Allocentric neglect score was adjusted from Rorden *et al*.^41^ and defined as the density of the differential error rate between distractors (opened apples) on the contralesional side (Contra_err) versus the ipsilesional side (Ipsi_err), normalized by the total number of correctly identified targets (Whole_corr), *A* = (Contra_err − Ipsi_err)/Whole_corr. The scores were analysed both in their absolute value and, as a control, in their original signed values, where positive and negative values corresponded to left-lateralized and right-lateralized deficits, respectively. These scores assess an egocentric lateralized inattentional bias and an object-based allocentric lateralized inattentional bias, respectively.

In the ‘clock drawing test’,^37^ patients were asked to draw the hours of a clock from memory on an A4 paper where the clock contour was already drawn. From these drawings, we obtained two different measurements. First, we calculated the left-to-right asymmetry of hour omissions or translocations (adapted from Ronchi *et al.*^42^). In brief, we assigned a value of 1 for each omission or left-to-right translocation of an hour from the right- or left-hand-side quarters of the quadrant; ‘12’ and ‘6’ were scored as left-to-right translocation when displaced in the right- or left-hand side outside a 30° angle centred on the correct location. We assigned a value of 0.5 for each translocation of the remaining hours beyond a 30° angle centred on the proper location.^42^ These values were separately calculated on each side of the clock, and the deficits were expressed as left-to-right asymmetry. In addition, we calculated a clock-margin asymmetry score by calculating the left-to-right difference in the distance between the clock circumference and the leftmost/rightmost hour mark, in order to capture asymmetries in perceived spatial extent and relationships within an object (Fig. 1B, bottom images). These scores assess a lateralized within-object spatial bias in the visuomotor encoding of a complex multipart object.

Two items from the ‘MoCA battery’^43^ were also included in our analysis, namely the cube/chair copy-drawing test and a separate clock drawing from memory. The first requires the participant to correctly copy-draw a three-dimensional object (cube or chair), and one point is assigned for correct drawings. The second requires the participant to draw from memory a clock on a blank sheet with its three components (contour, hands and numbers), and 0–3 points are assigned if none, one or more elements are correctly drawn. MoCA scores were converted so that a higher score corresponded to a stronger impairment to match the convention of the other tests. These scores assess spatial representation and visuoconstructional deficits. Importantly, we quantified the proportion of constructional deficits that could be attributed to lateralized impairments, in order to assess the extent to which these measures might reflect spatial neglect rather than pure constructional deficits. In our sample, 13% of patients (10 out of 77) exhibited signs of lateralized inattention in the chair/cube or MoCA-clock drawing. Control principal component analysis (PCA) showed that the exclusion of these patients from the sample did not result in a major change in the second component related to constructional deficit (Supplementary Fig. 1).

### Behavioural data analyses

The tests above collectively provided eight different scores related to visuospatial processing. Three scores are designed to probe for lateralized bias in attention and spatial exploration but covering different spatial scales and different levels of target/distractor ratios (Apples-egocentric, Bisection-5 cm and Bisection-20 cm); please note that while the first is widely recognized as a measure of egocentric deficits, the two line bisection tasks may reflect a combination of both egocentric and allocentric components.^44^ One score probed mainly for within-object allocentric attentional deficit (Apples-allocentric), whereas two other scores assessed lateralized biases in within-object spatial representation (clock-asymmetry and clock-margin), and two scores assessed more directly constructional deficit (from MoCA tests). It is important to note that, despite the apparent similarity between the two clock drawing tests, they differ in scoring criteria, task instructions and visuospatial demands. We therefore included both of them to verify whether these differences would engage distinct cognitive dimensions.

### Lesion and disconnection mapping

To draw the lesion maps, we used the brain images collected in the acute phase post-stroke as part of the routine clinical investigation after the patient was admitted to the hospital due to the onset of stroke symptoms, except in one case where the high-quality images were acquired a few months after the stroke. All 77 patients were investigated with MRI. Depending on the time delay between the symptoms’ onset and MRI scanning, the lesion was most clearly demarcated in the diffusion-weighted (62 cases), T2-weighted (14 cases) or T1 brain scans (1 case).

The lesion was mapped with the Clusterize toolbox, through the first automated identification of the local lesion clusters on each image slice based on its intensity, followed by subsequent manual validation and potential freehand correction.^45^ The resulting lesion map was then normalized to the Montreal Neurological Institute (MNI) single-subject template with the aid of SPM12 software (http://www.fil.ion.ucl.ac.uk/spm/). We applied to each map a deformation field estimated from the registered diffusion, T1 or T2. The mapping of lesioned brain regions of each patient is shown in Supplementary Fig. 2. The acute and chronic patient groups included a similar proportion of individuals with right-sided and left-sided lesions (acute *N* = 24/16; chronic *N* = 25/12). To collectively analyse acute and chronic populations and increase the statistical power of identifying a lesion pattern with a significant contribution to visuospatial deficits independent of the lesioned hemisphere,^46^ we mirror-flipped the left-lesioned hemisphere to the right along the centre line in MNI space using a custom-made script in Python 3.9.16 (https://www.python.org/downloads/release/python-3916/), as previously done in the literature.^47-49^  Supplementary Fig. 3A shows lesion overlaps for acute and chronic patients, respectively.

Finally, we used the Lesion Quantification Toolkit^50^ to determine—for each subject—three additional measures of brain damage: (i) an anatomo-functional measure of the lesion in grey matter and subcortical nuclei as parcelled by the Human Connectome Project-extended (HCPex) atlas, i.e. percentage of parcel damage,^51,52^ (ii) the percentage damage of the main longitudinal white matter tracts and (iii) the percentage damage of area-to-area disconnection^53-55^ constructed from the same HCPex diffusion-MRI (dMRI) datasets.

### Brain damage-symptom mapping

After determining for each patient the presence and extent of regional and disconnection lesions, we conducted four analyses to relate lesion sites and behavioural symptoms across our subpopulations of chronic and acute patients. The four analyses were meant to address the contribution of damage at different levels of brain functions, i.e. at the level of individual voxels (small population of neurons), at the level of anatomo-functional areas or parcels (e.g. visual area 1 or lateral intraparietal ventral area), at the level of large anatomical tracts serving multiple brain areas and at the level of area-to-area disconnection.

#### Voxel-based support vector regression-LSM

We employed support vector regression-LSM (SVR-LSM) analysis to map the behavioural components identified by the factorial analysis onto lesioned voxels.^56^ SVR-LSM is a development of multivariate LSM (MLSM) that relies on the support vector machine (SVM) method^57^ and can better identify complex dependencies between voxels, while showing high sensitivity and specificity for detecting lesion-symptom relationships.^56,58^ We conducted SVR-LSM analysis using the svrlsmgui toolbox (https://github.com/atdemarco/svrlsmgui) on MATLAB R2018b (https://mathworks.com/downloads). A linear regression was performed on binary brain lesions (Supplementary Figs 2 and 3), and an SVR model was used to predict the behavioural data,^57^ i.e. principal components estimated by the independent, data-driven factorial analysis (Fig. 1B). We regressed lesion volume out of both behaviour and lesion data prior to SVR.^16,57,59,60^ Traditional methods of lesion volume control^16,61,62^ typically involve regressing lesion volume from behavioural scores or lesion maps alone. However, they often leave unlesioned voxels uncorrected due to their zero values being unaffected by the transformation. The approach we adopted is more comprehensive: it regresses lesion volume from behavioural scores, from lesion maps and from both combined. This method is conceptually robust and practically effective, as it aligns closely with how covariates are typically handled in between-group statistical analyses (e.g. ANOVA) and in regression-based mass-univariate LSM tools such as vlsm2.^57^ To guarantee robust and stable results, we defined a threshold so that discrete lesions present in <10% of the participants were excluded when overlapping lesion images.^63-65^ We thus obtained SVR-*β* maps and corrected *z-*maps reflecting the brain areas whose damage correlated with each of the identified factors. We controlled for age, gender and time post-stroke as covariate variables through nuisance models. We also conducted control analyses on the right-sided patients only to assess consistency and similarities in the results.

#### Atlas-based lesion- and disconnection-symptom mapping

To identify relations between symptoms and major anatomo-functional areas, we correlated the percentage of parcel damage for each parcel defined by the HCPex atlas^51,52^ for each patient with the behavioural components identified by our prior factorial analysis (Fig. 1B). Similarly, by using a custom-made Python script with the same hyperparameters from the svrlsmgui toolbox, we conducted SVR-LSM on (i) the percentage damage of each parcel, (ii) the percentage disconnection of each anatomical tract and (iii) the percentage disconnection between each pair of parcels to obtain the association of symptom-parcel damage and symptom-anatomical tract disconnection. To guarantee robust and stable results, we defined a threshold so that parcels/tracts present in <10% of the participants were excluded when overlapping lesion images. We controlled for age, gender and time post-stroke of patients as covariate variables through nuisance models.

### Statistical analysis

To assess differences in the distributions of neuropsychological test scores between acute and chronic patient subgroups, as well as between right- and left-lesioned patients, we calculated the cumulative proportion of patients across score ranges and applied the Kolmogorov–Smirnov test^66^ to evaluate distributional differences.

**Figure 2:**
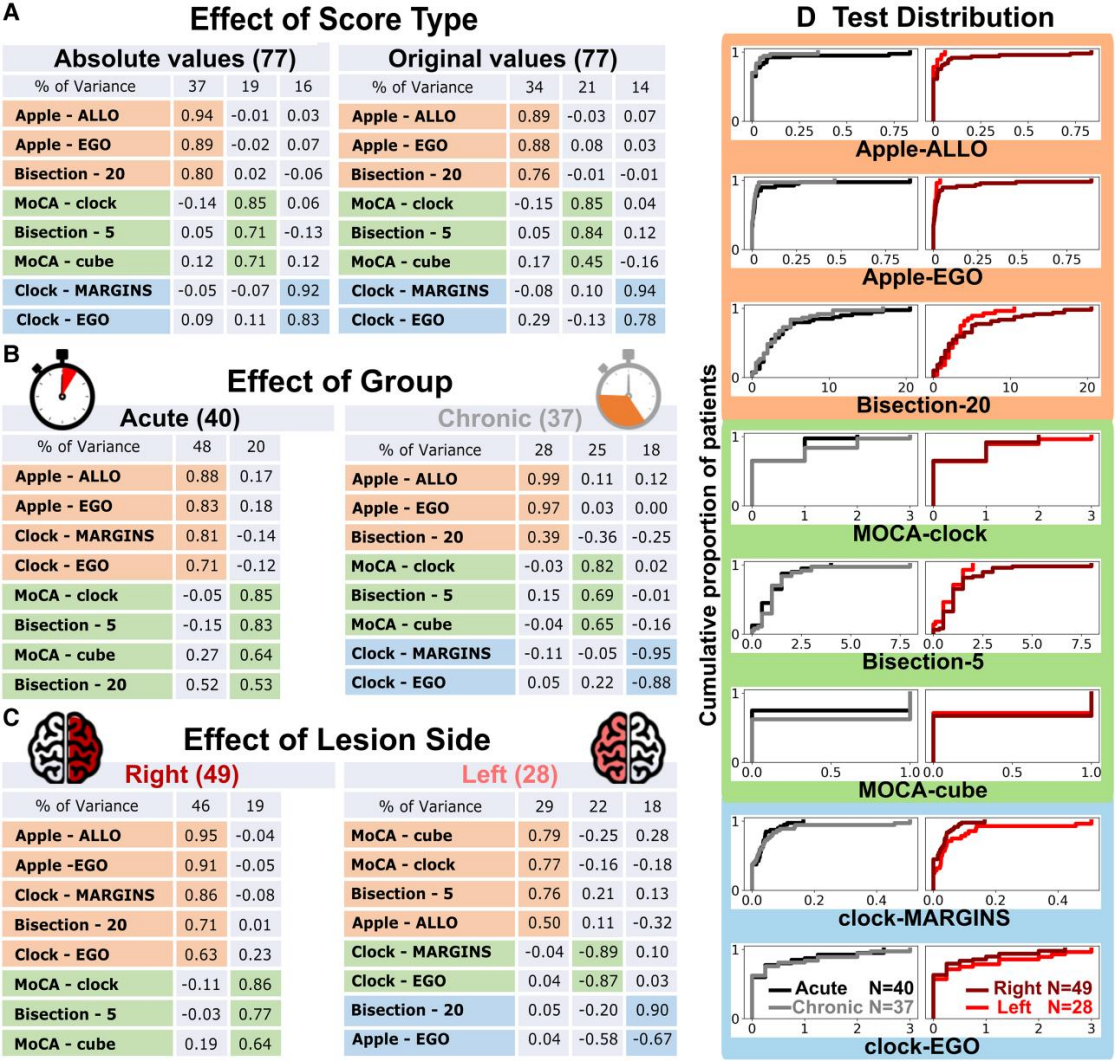
**Control analyses of PCA regrouping across score types and subpopulations and subgroup performance distributions.** (**A**) Control PCA testing the effect on regrouping of using the test absolute values (i.e. quantifying a bias but not the direction of the bias; left table) or the original test scores (i.e. with positive and negative signs quantifying the leftwards—negative—or rightwards—positive—direction of the bias; right table); here and below each table reports the principal components, the amount of variance explained, the eigenvalues and the factor loadings obtained by factor analysis of different score types and population subgroups. (**B**) Control PCA assessing regrouping separately in the acute (left table) and chronic (right table) populations. (**C**) Control PCA assessing regrouping separately in the right-lesioned (left table) and left-lesioned (right table) populations. (**D**) Empirical cumulative distributions of the eight neuropsychological tests separated for the population subgroups of acute (black) and chronic (grey) patients (left column) and of the two orthogonal populations of the right-lesioned (dark red) and left-lesioned (light red) patients (right column). Kolmogorov–Smirnov tests revealed no significant distributional differences between (black) and chronic (grey) patient groups, nor between right-lesioned (dark red) and left-lesioned (light red) patients (right column), across all neuropsychological assessments (*P* > 0.05, with *D*-statistics ranging from 0.04 to 0.3). PCA, principal component analysis; Apple, apple cancellation test; ALLO, allocentric; EGO, egocentric; Bisection-20, line bisection test in 20 cm length; MoCA-clock, Montreal Cognitive Assessment-clock drawing test; Bisection-5, line bisection test in 5 cm length; MoCA-cube, Montreal Cognitive Assessment-cube copying test; Clock, clock drawing test.

To extract shared cognitive dimensions across neuropsychological tests, a factorial analysis was performed on the eight scores (absolute value) from all patients (chronic and acute). PCA, i.e. a multivariate statistical technique used for dimensionality reduction, was calculated using a standard procedure with oblimin rotation and Kaiser normalization in the Statistical Package for the Social Sciences 26.0 (SPSS Inc., Chicago, IL, USA). We selected the most significant factors based on the amount of variance explained (Fig. 1). Then, we extracted the relative contribution (loading) of each test score to these factors and the relative magnitude of each factor for each patient. The latter values were used for all subsequent lesion analyses described below. To gain more in-depth knowledge about patterns of behavioural deficits among patients, we performed additional factorial analyses to assess whether (i) using absolute or signed scores, (ii) distinguishing between acute and chronic stages or (iii) considering lesion sides (left versus right) separately would organize behavioural deficits differently and affect subsequent mapping results (Fig. 2A and B).

To assess differences in brain damage between subpopulations of acute and chronic patients, we submitted the four lesion quantification indices (voxel-based, parcel-based damage, tract disconnection and parcel-to-parcel disconnection) to separate Mann–Whitney U-tests^67^—which compute differences between two groups on a single, ordinal variable with no specific assumption on the distribution. Voxel-based, atlas-based and disconnection-based SVR-*β* maps were computed to assess the relationship between lesion status and behavioural outcomes. Statistical significance was determined using permutation-based family-wise error (FWE) correction with 5000 permutations. For voxel-based SVR-LSM, we applied a voxel-level threshold of *P* < 0.005 (one-tailed) and a cluster-level threshold of *P* < 0.05 to correct for multiple comparisons.^57^ For atlas-based and disconnection-based SVR-LSM analyses, multiple comparisons were controlled using permutation-based FWE correction at *P* < 0.05 (one-tailed).^57^

## Results

Our population of stroke patients (Fig. 1A) included 40 with acute (24 right-hemisphere) and 37 with chronic (25 right-hemisphere) lesions. Nineteen presented signs of USN (9 acute/16 right-brain damage) and 33 of constructional apraxia (15 acute/12 right-brain damage), with a defective performance on at least one neuropsychological task, based on normative data. We first compared visuospatial symptoms in either the acute or chronic post-stroke phase and then examined which brain areas and connections may be associated with distinct patterns of deficits.

### Lateralized inattention and constructional deficits reflect acute versus chronic stages and right versus left lesions

We ran a factorial analysis on eight visuospatial neuropsychological test scores (Fig. 1B, *Test overview*) across all patients (acute and chronic) and obtained three significant factors explaining 72% of the total variance (Fig. 1B, *PCA values*). Different clusters of tests were distinguished as a function of their loadings on specific PCA components. The first component (Fig. 1B, orange area) regrouped performance on apple cancellation, including both egocentric and allocentric score biases, together with long line bisection (20 cm) deviation. The second factor (Fig. 1B, green area) regrouped two scores reflecting constructional impairment in drawing (cube and clock from the MoCA battery) and deviation on bisection of short lines (5 cm). The third factor (Fig. 1B, blue area) regrouped the clock-margin and clock-asymmetry scores. Interestingly, although two measures entail a similar task (clock drawing from MoCA and from neglect battery), their scorings aim to tap into different visuospatial abilities and consistently dissociate into two different components in our factorial analyses (see below). Similarly, while both Components 1 and 3 regroup scores representing lateralized biases, the first concerns tests with multiple stimuli and distractors on a full paper page (cancellation test) or a larger stimulus (line bisection), requiring wider exploration and filtering of irrelevant inputs, while the third mostly concerns tests with a single smaller object with internal elements placed in a more global spatial configuration. The results remained consistent in a control analysis where we excluded the 5 cm line bisection test (Supplementary Fig. 1A), thereby ruling out potential confounds related to the unequal availability of normative data for the two line bisection tests,^37,38^ as well as the possible influence of a crossover effect that may occur with shorter lines.^68^ A further control PCA excluded patients who showed neglect-like signs in the constructional MoCA tasks. These results (Supplementary Fig. 1B) reinforce our previous findings by showing that the second component reliably emerged even after accounting for potential confounds associated with different stimulus types. These results were also consistent across factorial analyses where we inputted either the original test scores or their absolute values (i.e. quantifying a bias but not the direction of the bias) (Fig. 2A), highlighting their robustness across different metrics. We interpret these three factors as reflecting visuospatial attention and between-object exploration, visuoconstructive ability and within-object spatial organization, respectively (Fig. 1B, rightmost column).

To understand how the two subpopulations of acute and chronic patients specifically contributed to these results, we analysed the empirical cumulative distributions of each score in each group (Fig. 2D, black versus grey distributions). Although not significant, the acute population showed higher values (more impaired) in the first three tests regrouped in Component 1, while the chronic population showed higher scores (more impaired) in the three tests regrouped in Component 2. The chronic population was also slightly more impaired in the clock-margin tests, while the egocentric bias of the clock drawing gave virtually identical cumulative distributions. Interestingly, when we considered an orthogonal regrouping of the whole population into right-lesioned and left-lesioned patients, we found similar results for the first two components (Fig. 2D, dark red versus light red distributions): although not significant, the right-lesioned population showed higher values (more impaired) in the lateralized spatial attention tests, while the left-lesioned population showed higher scores (more impaired) in the constructional tests. For the third component, the right-lesioned population showed higher values (more impaired) in the single-object tests.

These distinct functional components were further dissected by two additional factorial analyses run independently in each of the two subpopulations of acute and chronic patients (Fig. 2B). Remarkably, each factorial analysis returned similar factors but with important differences. In the acute population, the first component, i.e. the one explaining most of the variance (48%), regrouped lateralized bias scores from both the cancellation test (large between-object space) and the clock drawing test (narrow within-object space). The second component, which explained less than half of the variance (20%), loaded on the constructional and bisection tests. In contrast, in the chronic population, the three components regrouped the same tests as those found for the whole sample, including (i) both local and global spatial deficits in the cancellation and long line bisection tests, (ii) constructional impairments in drawing tasks and deviation in short line bisection and (iii) more local/internal object organization in the clock task. However, the first and second components explained a similar amount of variance, confirming the higher contribution of constructional deficits in the chronic population previously shown by the cumulative distributions (Fig. 2D, left column).

When we subgrouped the stroke population into right-lesioned and left-lesioned patients, another interesting distinction appeared. In the right-lesioned population (Fig. 2C), the first component, i.e. the one explaining most of the variance (46%), regrouped the lateralized bias scores from the cancellation test, the clock drawing test and the long line bisection test, while the second component, explaining less than half of the variance (19%), loaded on the constructional tests and short bisection. On the contrary, in left-lesioned patients, the first component regrouped the constructional deficits and short line bisection together with the allocentric component of cancellation (though with a lower loading factor), while the second component loaded on the two clock drawing scores, and the third component regrouped the two global scores (apple cancellation and long bisection), each accounting for a much lower variance (18%).

Overall, this pattern of results shows that visuospatial deficits tend to regroup into three main factors across different subpopulations of stroke patients, and while for acute and right-lesioned patients lateralized spatial biases in attention and exploration are dominant, for chronic and left-lesioned patients visuospatial constructional deficits play a more important role. These differences suggest a double dissociation of underlying functional components across the four subgroups (acute versus chronic and right- versus left-lesioned).

### Voxel-based LSM shows distinct brain patterns for the three behavioural components in the acute and chronic populations

**Figure 3:**
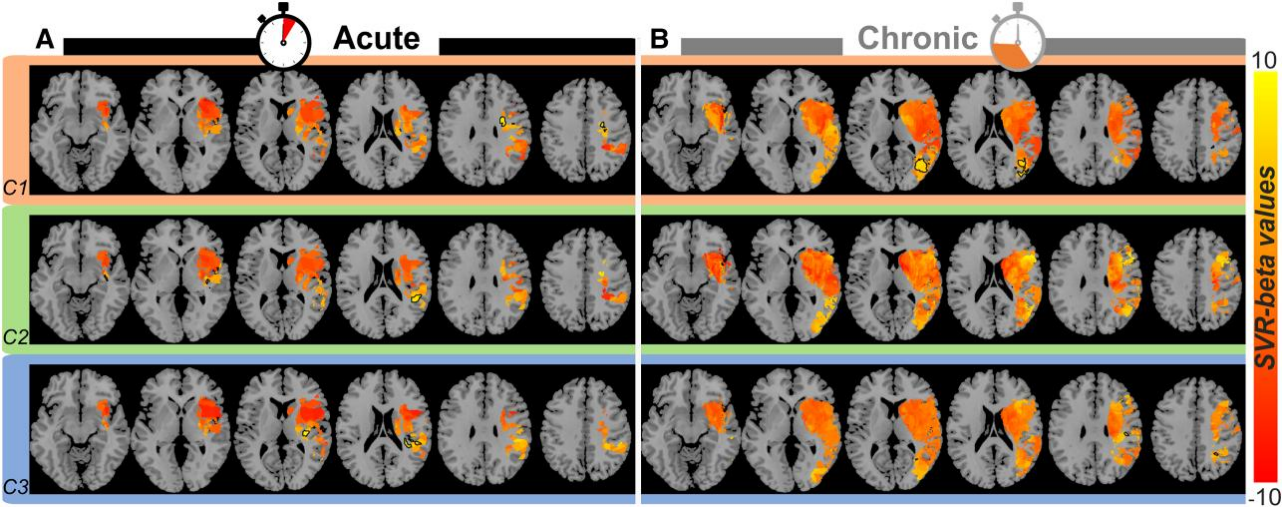
**Voxel-based LSM for the acute and chronic populations.** Beta value results from the SVR model for the three behavioural components of deficits in the acute (**A**) and chronic (**B**) populations; left lesions have been flipped to the right hemisphere (see ‘Materials and methods’ section); black contours show the voxels that survived the permutation-based (*n* = 5000) FWE correction (*P* < 0.005, one-tailed). C1, Component 1; C2, Component 2; C3, Component 3; SVR, support vector regression.

To identify the brain structures involved in different visuospatial deficits, we applied SVR-LSM to the sample of patients using their continuous scores assigned by the PCA (Fig. 1B). Acute and chronic populations exhibited similar patterns of damage (Supplementary Fig. 3). Specifically, the voxel-based (Supplementary Fig. 3A), parcel-based (Supplementary Fig. 3B and C) and disconnection-based (Supplementary Fig. 3D and E) lesion patterns were largely comparable between the two populations, and we did not find any significant difference in lesion volume size (U = 556.0, *P* > 0.05), parcel damage percentage (U > 561.5, *P* > 0.05) or tract-based disconnection percentage (U > 453.5, *P* > 0.05) between acute and chronic groups after Bonferroni correction for multiple comparisons^69^; the chronic population showed a generally higher degree of damage, but differences in lesion global anatomical distribution were not significant. Despite the similarities of the lesion maps in the populations of acute and chronic patients (Supplementary Fig. 2), their correlation with behaviour was substantially different (Fig. 3).

In the acute group, the lateralized global/local inattention component (Fig. 3A, orange areas) was associated to lesions in the superior and middle frontal gyri, with a main peak located in white matter near the precentral gyrus (MNI coordinates: 29, −1, 28), plus a few other smaller peaks between precentral–postcentral regions (MNI: 30, −26, 37 and 43, −6, 30). Constructional deficits (Fig. 3A, green panel) were instead mainly associated with two distinct peaks in the posterior superior temporal cortex (MNI: 53, −37, 9 and 48, −30, 3) and in the white matter extending to supramarginal and middle temporal regions. A small cluster in the posterior insular cortex and into adjacent white matter was also found (MNI: 38, −20, −2). Finally, for the lateralized within-object spatial component (Fig. 3A, blue panel), statistically significant lesion peaks were observed in regions near those above, including the posterior superior temporal and supramarginal gyri extending more dorsally towards the parietal lobe (MNI: 48, −37, 16), as well as in the white matter underneath the insula (MNI: 38, −13, 5) and rolandic operculum (MNI: 32, −32, 15). In addition to these differences, we observed a partial overlap of lesion sites in white matter regions between the thalamus and the putamen for the first and third components, which may be related to their less differentiated behavioural profile seen in the factorial analysis (Fig. 2B, acute; where lateralized inattention and lateralized within-object bias belong to the same component). We also observed partial overlap of lesion sites for the first and second components, which may be related to an influence of USN on constructional scores. Furthermore, a notable overlap of lesion sites was observed between Components 2 and 3, possibly linked to their object-based nature.

In the chronic group, the lateralized global/local inattention component (Fig. 3B, orange panel) was mainly associated to posterior lesions in the middle temporal, middle occipital and angular cortex, with extension into adjacent white matter (MNI: 37, −63, 8), plus a smaller peak in the inferior parietal lobe (MNI: 29, −47, 50). The constructional component was associated with a more distributed network with clusters in the inferior and middle gyri and adjacent white matter, posterior temporal and middle occipital areas, as well as in the angular gyrus (Fig. 3B, green panel). Mapping of the lateralized within-object component was also multifocal (Fig. 3B, blue panel) and implicated the inferior parietal cortex, superior parietal lobule and intraparietal sulcus (MNI: 40, −55, 54). A peak in white matter adjacent to lower postcentral regions was also found (MNI: 47, −24, 28). Similar to the second component, a high correlation was found in the occipitoparietal cortex, possibly linked to the object-based features of this component.

Due to the complex interplay between acute and chronic deficits (Fig. 2C) and the presence of both right and left lesions in preceding analysis (Fig. 3), we conducted additional LSMs in right-lesioned patients only (Supplementary Fig. 4), in whom visuospatial disturbances are most frequently reported. In the acute group, the first and third components relied on more distributed networks, while the second showed a more limited number and extension of significant peaks (Supplementary Fig. 4A versus C), in line with behavioural results showing a lower presence of constructional deficits in these patients compared to left-lesioned ones (Fig. 2C, right panel). In the chronic group, also, the first and third components mapped onto more distributed networks, with reduced correlations for the first component (Supplementary Fig. 4B versus D, orange areas), in line with behavioural results showing weaker lateralized inattentional deficits in the chronic phase. Interestingly, the second component showed a much more distributed network and stronger correlations (Supplementary Fig. 4A versus D, green areas), also consistent with a higher rate of constructional deficits (Fig. 2B, right panel).

Overall, voxel-based LSM in the acute group revealed that lesions associated with lateralized inattention, constructional deficits and within-object spatial processing were primarily located in regions such as the frontal gyrus, superior temporal cortex and posterior parietal lobe. In the chronic group, similar deficits were instead linked to lesions in the posterior temporal, occipital and angular cortex, as well as the insular and parietal regions. Additionally, overlapping lesion sites between Components 2 and 3 suggest shared involvement in object-based processing.

### Atlas-based region and disconnection mapping reveals distinct brain lesion-symptom patterns in acute and chronic phases

Voxel-based SVR-LSM analyses allow pinpointing the relationship between damage at any given voxel and different levels of cognitive deficits without incorporating information neither about the functional modules in which the grey matter is organized, nor about the distinction between grey and white matter, and in particular about the disconnection of functional modules. We therefore ran SVR-LSM on the grey matter and subcortical nuclei parcellation provided by the HCPex atlas^51,52^ (Supplementary Fig. 3B and C) and the white matter parcellation provided by the Yeh atlas^70^ (Supplementary Fig. 3D and E), for the three major deficit components in the acute and chronic populations.

**Figure 4:**
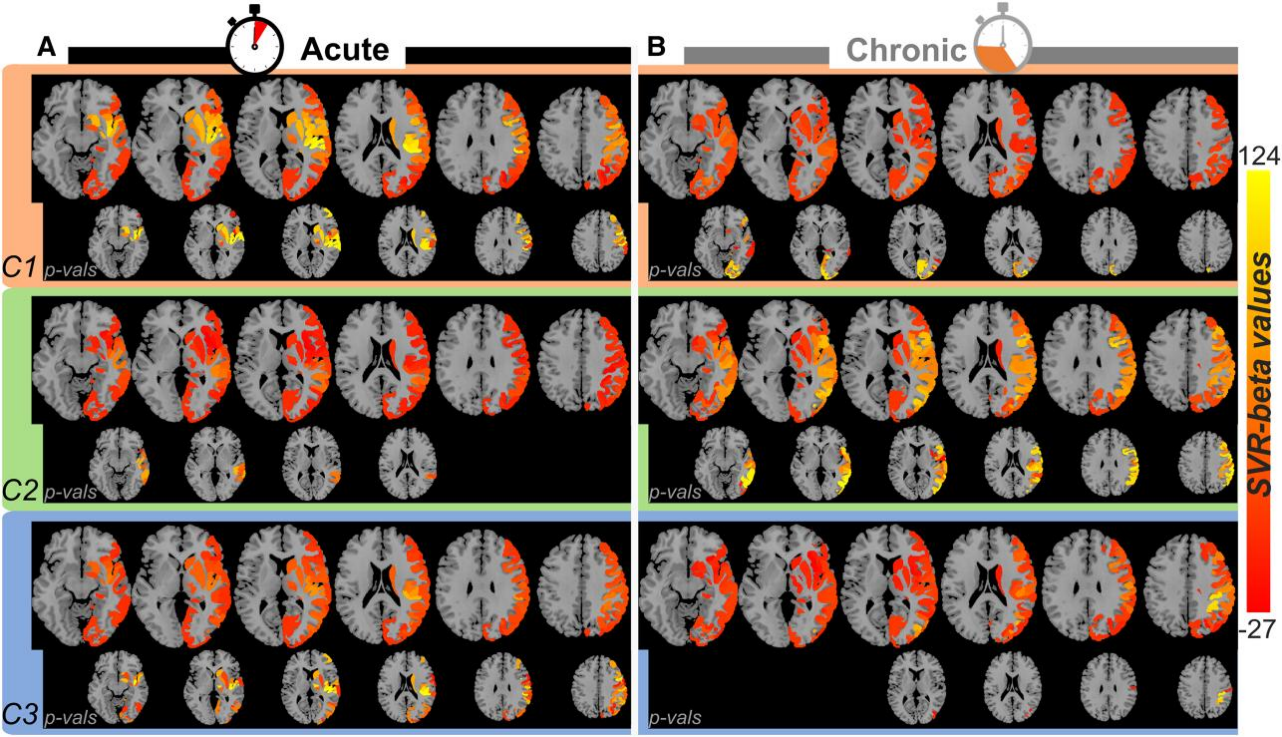
**Parcel-based LSM.** (**A**) Acute group and (**B**) chronic group. The SVR-beta value colour bar indicates the correlation strength between the damage of each parcel and the three components, which is projected back to the brain parcels. Insets show corresponding significance levels (1 − *P*) of all parcels that survived after the permutation test (*n* = 5000), thresholded at *P* < 0.05, uncorrected, with red representing the most significant parcels and blue the least significant parcels. Upper panels/orange: lateralized global/local inattention; middle panels/green: constructional component; lower panels/blue: lateralized within-object component; uncorrected *P-*value colour bar indicates the correlation strength that survived after the permutation test (*n* = 5000, uncorrected). All the results survived after 5000 permutation tests (uncorrected). Damage of FEF (*P* = 0.04) and PEF (*P* = 0.02) related to the lateralized inattention component in the acute group survived after FWE correction with *P* < 0.05 based on 5000 permutations. C1, Component 1; C2, Component 2; C3, Component 3; SVR, support vector regression.

At the level of anatomo-functional parcels, we found that in the acute group, the lateralized global/local inattention component (Fig. 4A, orange area) was associated with damage in sensorimotor, mid-frontal, insula and subcortical regions, with significant correlations observed especially in the frontal eye field (FEF) and prefrontal eye field (PEF) (detailed parcel list in Supplementary Table 2). Both the constructional (Fig. 4A, green area) and lateralized within-object components (Fig. 4A, blue area) were associated with lesions in the sensorimotor, auditory and posterior insula regions, extending to the inferior parietal lobe with a peak around the angular gyrus for the former and more superior temporal area for the latter. In the chronic group, the lateralized global/local inattention component (Fig. 4B, orange area) was associated with damage in high-level extrastriate visual areas in the posterior temporal and inferior lateral occipital cortex. The constructional component (Fig. 4B, green area) was associated with both the dorsal and ventral stream visual regions, such as the V4, lateral occipital complex, middle temporal complex and temporo-parieto-occipital junction. Finally, the lateralized within-object component (Fig. 4B, blue area) was associated selectively with dorsal stream visual parcels in the superior parietal cortex (anterior intraparietal area). Overall, these data show consistent results across both voxel-based and parcel-based LSMs. The strength of parcel-based correlations seemed to better reflect the quantified behavioural deficits (stronger lateralized inattention in the acute phase and stronger lateralized inattention and stronger constructional deficits in the chronic phase), both in terms of beta values and *P*-values, likely because parcel-based mapping more effectively captures functionally defined modules of the brain. The acute group seemed to show slightly more differences across the voxel-based versus parcel-based analyses, especially for the lateralized inattention component, possibly suggesting a higher contribution of disconnections that could not be captured by any parcel by definition.

**Figure 5:**
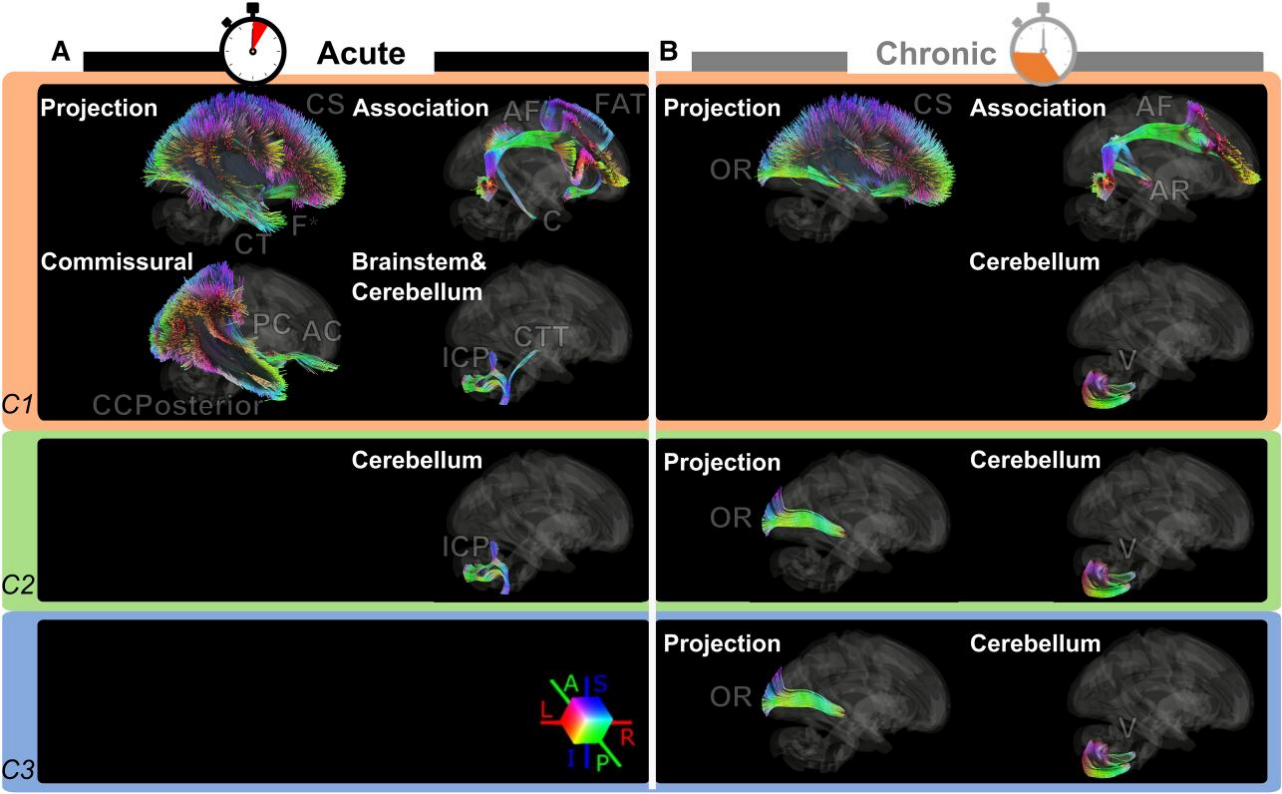
**Tract-based LSM.** (**A**) Acute group and (**B**) chronic group. Each panel shows the connectome tracts associated with the three behavioural components: lateralized global/local inattention (orange), constructional component (green) and lateralized within-object component (blue). All tracts shown above survived after the permutation test (*n* = 5000), thresholded at *P* < 0.05, uncorrected. F survived after multicomparison correction (*P* < 0.05, FWE corrected) based on 5000 permutations. CS, corticostriatal; CT, corticothalamic; F, fornix; AF, arcuate fasciculus; C, cingulum; FAT, frontal aslant tract; AC, anterior commissure; CCPosterior, posterior part of the corpus callosum; PC, posterior commissure; CTT, central tegmental tract; ICP, inferior cerebellar peduncle; OR, optic radiation; AR, acoustic radiation; V, vestibulocerebellar; C1, Component 1; C2, Component 2; C3, Component 3; SVR, support vector regression; A, anterior; P, posterior; L, left; R, right; S, superior; I, inferior.

At the level of major tract disconnection (tract-based SVR-LSM), we found that in the acute group, the lateralized global/local inattention component (Fig. 5A, orange area) was associated to a disconnection of several cortico-cortical association tracts (arcuate fasciculus, cingulum and frontal aslant tract), commissural tracts (anterior commissure, posterior part of the corpus callosum and posterior commissure) and subcortical projection tracts (corticostriatal, corticothalamic and fornix) as well as the brainstem and cerebellar tracts (central tegmental tract and inferior cerebellar peduncle). The constructional component (Fig. 5A, green area) was associated only to a disconnection of the inferior cerebellar peduncle, while the lateralized within-object component was not associated with any consistent tract disconnection. In the chronic group, lateralized inattention (Fig. 5B, orange area) was associated with disconnections of two association tracts (arcuate fasciculus and acoustic radiation), two projection tracts (corticostriatal and optic radiation) and one cerebellar tract (vestibulocerebellar). Interestingly, the constructional component (Fig. 5B, green area) was associated with disconnections of the optic radiation and vestibulocerebellar tract, while the lateralized within-object component (Fig. 5B, blue area) showed a similar but weaker association with the same tracts.

Overall tract-based LSM in the acute group indicates a central role of widespread disconnection for the lateralized inattention component, including interhemispheric callosal fibres. Importantly, while the parcel-based analysis highlighted differences between the second and third object-based components, the tract disconnection analyses revealed important similarities instead, especially in the chronic group. More precisely, visuospatial deficits for object-based components in this group were associated to the optic radiation, pointing to a possible contribution of disconnections in the ventral visual stream.

**Figure 6:**
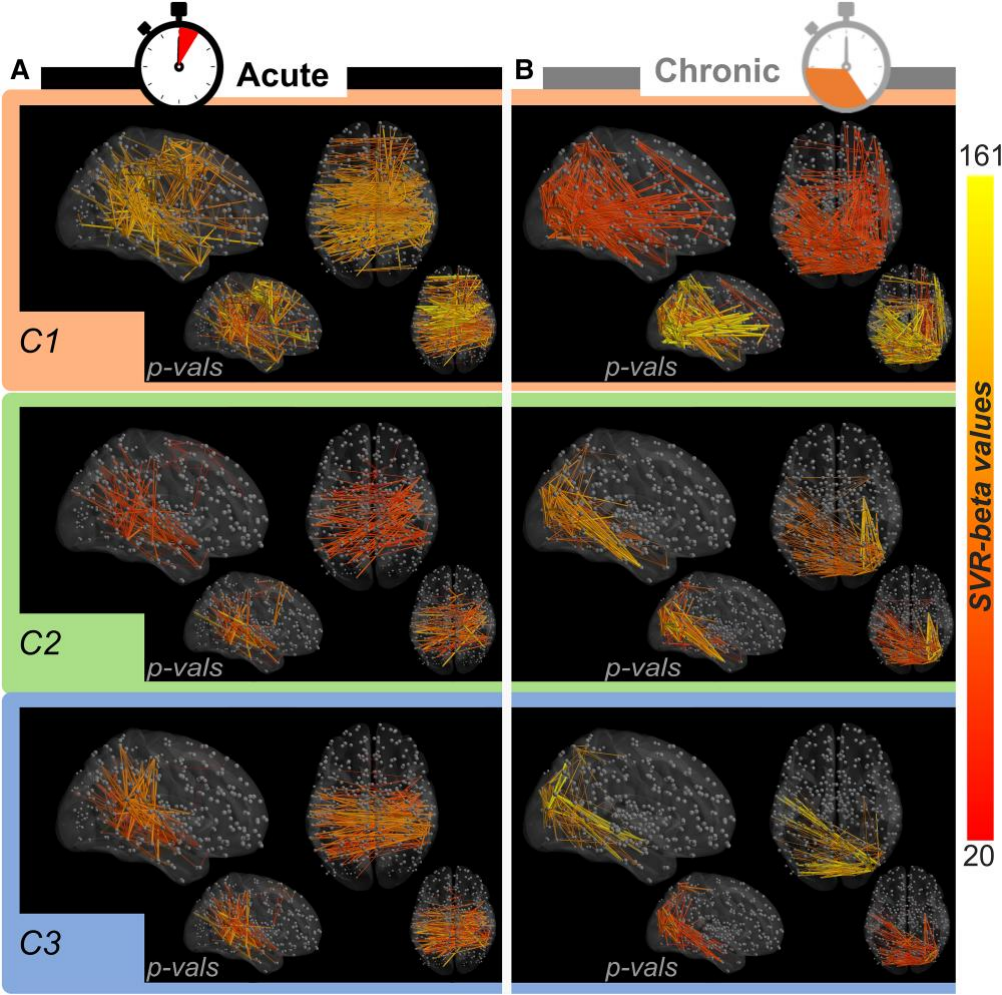
**Parcel-to-parcel disconnection LSM.** (**A**) Acute group and (**B**) chronic group. Each circle (node) indicates a parcel from the HCPex atlas, and each line (edge) indicates a disconnection between the two parcels [*n*_node_ = 426; *n*_edge_ = *n*_node_ × (*n*_node_ − 1)/2]. The edge colour indicated the beta values resulting from the SVR-disconnection-symptom mapping, as indicated by the colour bar. All disconnections survived after the permutation test (*n* = 5000), thresholded at *P* < 0.05, uncorrected. Insets show corresponding significance levels (1 − *P*), with red representing the most significant parcels and blue the least significant parcels. C1, Component 1; C2, Component 2; C3, Component 3; SVR, support vector regression.

Finally, to improve the resolution of the disconnection-symptom mapping, we analysed parcel-to-parcel disconnections. These results confirmed a strong impact of disconnections for the acute lateralized inattention component and further highlighted an important role of both interhemispheric disconnections (Fig. 6A, orange area) and disconnections of FEF and PEF (Supplementary Table 3), consistent with the parcel-based analysis in Fig. 4A. A similar but more posterior disconnection pattern was found in the chronic group (Fig. 6B, orange panel). For the constructional component, the parcel-to-parcel analysis revealed a disconnection of parietotemporal areas (Fig. 6, green area), with more posterior and inferior temporal areas of both the dorsal and ventral visual streams (Supplementary Table 3) in the chronic phase. Finally, the lateralized within-object component (Fig. 6, blue area) tapped on a similar pattern of disconnections with a more predominant involvement of temporoparietal and posterior interhemispheric communication. Overall, this parcel-to-parcel disconnection analysis complemented the parcel damage analysis by providing fine-grained disconnectivity patterns of functional modules. Like for the parcel damage analysis, the strength of parcel disconnection correlations seemed to well reflect the quantified behavioural deficits (stronger lateralized inattention in the acute phase and stronger lateralized inattention and stronger constructional deficits in the chronic phase), suggesting these analyses effectively capture white matter anatomo-functional disconnections.

## Discussion

Visuospatial impairments have a significant impact on patients’ lives after a stroke. In addition to well-known USN deficits, less-studied object-based deficits can also hinder recovery and overall well-being.^39,71^ Here, we provide a detailed characterization of a broader range of visuospatial deficits across different stages post-stroke that integrated information from different levels of brain functioning. Our primary objectives were to determine whether various visuospatial deficits persist over time and how they change from the acute to chronic stages, as well as to identify the brain structures involved.

To do so, we leveraged clinical neuropsychological tests assessing different components of visuospatial deficits, ranging from perceiving a single simple object (e.g. a line) to selecting a target across multiple different objects (e.g. selecting several full/incomplete apples) and assembling components within a single complex object (e.g. drawing a clock or a cube). The analysis of behavioural performance on these tests by means of dimensionality reduction methods revealed that the observed neuropsychological deficits regrouped into three main factors, which we interpreted as follows: (i) lateralized spatial attention biases (global or local), reflecting visuomotor exploration and selection of relevant targets among distractors, (ii) constructional deficits, reflecting the reproduction of coherent object configurations, and (iii) lateralized within-object biases, reflecting the proper organization of left-to-right spatial relationships of the object’s parts. In line with previous studies focusing on neglect, these results show the ability of dimensionality reduction methods to delineate factors that encompass key behavioural dimensions related to perceptive and exploratory abilities (e.g. Verdon *et al.*^10^ and Takamura *et al.*^72^). Moreover, they broaden this perspective to visuospatial behaviours such as constructional or within-object symptoms that are not necessarily related to an egocentric bias in spatial attention or awareness but still related to the proper perception and localization of objects or object parts in space.^27,73^

Crucially, by quantifying these deficits in two subpopulations of acute and chronic stroke patients, we demonstrated that lateralized attention deficits predominate in the acute phase, while constructional deficits are more prevalent in the chronic phase. Previous studies have focused on explaining the heterogeneity of spatial deficits associated with USN, typically examining either the acute, subacute or chronic phase, with some predicting and tracking the recovery of neglect over time.^17,39,74-80^ Here, we delve deeper into these notions by showing that although typical signs of USN may attenuate, other visuospatial deficits can persist. These findings suggest the potential for varying rehabilitation strategies over time and highlight the importance of a refined longitudinal perspective in the neuropsychological investigation of stroke patients.

Interestingly, we also observed a double dissociation between subgroups of acute versus chronic and right- versus left-lesioned patients. Acute and right-lesioned patients exhibited a predominance of biases in spatial attention, while chronic and left-lesioned patients showed more distinct constructional deficits. These findings are in line with a well-known lateralization of spatial and attentional processes^81^ and converge with a long-standing^82^ yet debated^83,84^ view of lateralization of constructional deficits, where left and right lesions produce different subtypes of constructional symptoms. In particular, left-lesioned patients may show the strongest deficits in drawing from memory tasks, as used in this study,^85^ and recover faster than right-lesioned patients.^86^ Our sample size was designed to include two comparable populations of acute versus chronic stroke patients, but the left-lesioned group was smaller in both cases, limiting our ability to conduct more in-depth analyses. Future studies should extend these findings to larger populations of patients with left- versus right-sided lesions. Additionally, to validate the observed evolution of cognitive deficits from the early to late post-stroke phases, future research could adopt a longitudinal approach, assessing the same patients at two different time points. Further, it would be useful to compare these clinical deficits to the persistence of other more precisely defined impairments, including not only attentional (re)orienting but also spatial working memory and cross-saccade remapping, which have been reported in both subacute and chronic neglect^80^ and might contribute to both lateralized deficits^87,88^ and non-lateralized constructional deficits.^23^

To pinpoint the brain regions and connections associated with these impairments through different phases of recovery, we used state-of-the-art machine learning-based mapping techniques. These were deployed at different levels of brain functioning, from local populations of neurons (voxel-based) to anatomo-functional areas (parcel-based) and from anatomical fibre bundles to fine-grained parcel-to-parcel disconnections. This methodological perspective has enhanced our understanding of a multivarious range of visuospatial deficits by providing detailed information about differential disconnection patterns previously unavailable in earlier studies.

Our findings reveal distinct neural correlates underlying visuospatial deficits in acute and chronic stroke populations, highlighting significant changes in the behaviourally relevant lesion-symptom and disconnection patterns over time. These differences may reflect the interplay between structural damage and functional deficits, neural plasticity and compensatory mechanisms, as well as the transition of such factors from acute to chronic phases.

Voxel-based LSM (SVR-LSM) demonstrated clear differences in brain regions associated with visuospatial deficits in acute and chronic patients. In the acute phase, deficits in global/local lateralized inattention were linked to lesions in the superior and middle frontal gyri, white matter near the precentral gyrus and posterior temporal and parietal areas. The parcel-based analysis also consistently pointed to precentral and premotor areas (and adjacent subcortical structures) and more precisely to FEF and PEF. This finding aligns with previous studies,^89^ especially pointing to a well-known role in selective attention for these areas,^90,91^ particularly its visuoexploratory component^10,92^ and resistance to distracting information.^87^ Constructional deficits were primarily associated with damage to posterior superior temporal regions, middle temporal regions and supramarginal gyri, consistent with earlier characterizations of constructional deficits in subacute patients.^23,24,93^ Lateralized within-object spatial processing deficits involved overlapping regions near the posterior temporal cortex and insula, with the addition of the putamen in the right-hemispheric patient group. These results are consistent with previous studies^11,94^ and highlight an important role of the temporal lobe in object-centred spatial biases. In the chronic phase, a shift in lesion-symptom relationships was observed, with global/local lateralized inattention linked more robustly to white matter damage in the posterior parietal lobe and with both grey and white matter in the superior occipitotemporal lobes. The posterior parietal cortex was more prominently implicated than the temporal cortex in the subpopulation of right-only patients. Constructional deficits were associated with a distributed network, including dorsal and ventral stream regions (e.g. the superior and inferior parietal lobes, the middle and inferior temporal lobes and the middle occipital lobes), as well as the posterior insula, a pattern aligning with previous studies using traditional voxel-based approaches.^78^ Within-object processing deficits involved dorsal stream regions such as the superior parietal lobule, angular gyrus and postcentral gyrus, with the latter playing a major role in right-hemispheric patients.^95^ These phase-dependent differences suggest a dynamic reorganization of the functional contributions of brain regions to visuospatial abilities, likely driven by neural plasticity and compensation mechanisms.

Tract-based and parcel-to-parcel disconnection analyses provided further insights into the white matter pathways and interregional connectivity disrupted in stroke patients. In the acute phase, lateralized global/local inattention was associated with a pervasive role of disconnection of cortico-cortical association tracts (e.g. arcuate fasciculus, cingulum and frontal aslant tract), commissural tracts (e.g. corpus callosum and anterior commissure) and subcortical pathways (e.g. corticostriatal and corticothalamic tracts), with a strong impact of interhemispheric disconnections particularly involving FEF and PEF as highlighted by parcel-to-parcel analysis. Although few previous studies have delved into this level of granularity, these data align with prior disconnection analyses^91,96^ and, in particular, with those highlighting interhemispheric disconnections,^97,98^ which could prevent a coherent spatial integration of parts into a full object. Constructional deficits were linked to a disconnection of the inferior cerebellar peduncle, potentially reflecting its role in local feature processing during drawing tasks,^93^ while a more fine-grained parcel-to-parcel disconnection analysis revealed additional contributions from occipitotemporal and temporoparietal disconnections. Lateralized within-object deficits showed minimal tract disconnection, possibly reflecting a more localized processing in these tasks, yet parcel-to-parcel disconnection suggested potential contributions of vertical parietotemporal and precentral–temporal disconnections that are typically less well captured by connectome-based parcellation approaches.^99^ In the chronic phase, lateralized inattention was associated with disconnections in ventral visual stream pathways, including the arcuate fasciculus and optic radiation, as well as commissural and cerebellar pathways, converging with previous work.^95^ Constructional and within-object deficits exhibited similar but more extensive disconnection patterns, especially in chronic phases, involving both dorsal and ventral visual streams, such as the optic radiation and temporoparietal tracts, and less interhemispheric disconnectivity. This suggests greater reliance on visual stream connectivity for object-based processing in chronic stages.

Overall, our findings suggest a higher involvement of frontal regions in the acute phase and a shift towards parietotemporal regions in the chronic phase for spatial attentional deficits, consistent with prior reports in right-lesioned patients^22,71,100^; this finding underscores the importance of examining lesion-symptom relationships across various populations at various phases post-stroke onset. For constructional deficits, even though many studies highlighted that figure drawing is a complex task relying on large-scale networks involved in both hemisphere,^97,101^ we found very few studies adopting a disconnection perspective (but see Nielsen^102^). Our results are therefore novel and clinically relevant and call for future investigations tapping into a more diverse exploration of constructional subtypes, beyond draw-from-memory tests. Future work should also further dissect components associated with spatial perceptual functions from those associated with motor and planning functions. Interestingly, lateralized within-object deficits relied on networks that were partly similar to those for constructional deficits, particularly in chronic patients. This similarity could account for some commonalities in the task loadings on these two components, presumably reflecting the role of perceiving and/or integrating the spatial relationship of different elements within a single object. These findings highlight the interrelated roles of lesions and interregional connectivity in visuospatial deficits and point to the importance of targeted rehabilitation strategies tailored to the specific lesion and disconnection patterns underlying chronic impairments.

## Conclusion

Our findings provide a nuanced understanding of the neural mechanisms underlying visuospatial deficits in stroke patients. The dynamic reorganization of lesion-behaviour and disconnection-behaviour relationships underscores the importance of addressing both grey and white matter damage in rehabilitation. Future studies could explore how these neural correlates evolve with therapy and recovery to optimize interventions targeting acute and chronic visuospatial deficits.

## Supplementary Material

fcaf477_Supplementary_Data

## Data Availability

Custom-made scripts for SVM analysis are available on GitHub: https://github.com/Jiesong2358/SVR-LSM. Anonymized demographic, behavioural and lesion data are available on the Open Science Framework (OSF): https://osf.io/4ewuc/?view_only=660f249e9721472ab89dd5fc9f4a6026.
