# Supplementary material for: From acute neglect to chronic constructional deficits: parietotemporal contributions to long-term post-stroke impairments
Source: Brain Commun. 2025 Dec 4;7(6):fcaf477. doi: 10.1093/braincomms/fcaf477 (PMC12704327; doi:10.1093/braincomms/fcaf477)
Supplement: fcaf477_Supplementary_Data [file fcaf477_supplementary_data.pdf]

Supplementary materials

From acute neglect to chronic constructional deficits: parieto-temporal contributions to long-term post-stroke impairments

Short title

From neglect to constructional deficits

Authors and affiliations

Jie Song[1], Eugenie Cataldo[1], Marine Thomasson[2], Arnaud Saj[1,3], Patrik Vuilleumier[1]*, Roberta Ronchi[1,2]*, Ilaria Sani[1]*[†]

1. Department of basic neurosciences, Faculty of medicine, University of Geneva, Switzerland
2. Department of clinical neurosciences, Hôpitaux Universitaires de Genève, University of Geneva, Switzerland
3. Departement de psychology, Faculty of arts and sciences, University of Montreal, Canada

* Patrik Vuilleumier, Roberta Ronchi and Ilaria Sani contributed equally to this work.

† = corresponding author

Ilaria Sani

Department of basic Neurosciences

Campus Biotech, Chemin des Mines 9, 1202 Geneva, Switzerland

Supplementary text

Acute and chronic populations show similar patterns of damage at the voxel, parcel, and disconnection levels.

The two populations of acute and chronic patients showed overall similar patterns of damage (Supplementary Figure 2), with a higher incidence of right-hemispheric rather than left-hemispheric lesions (24 vs. 16 and 25 vs. 12, see also Fig. 1A and Supplementary Figure 1). After lesion flip, the 40 acute patients (Supplementary Figure 2A, left panel) showed maximal lesion overlap in the deep territory of the middle cerebral artery, centered on the subcortical nuclei putamen, caudate, and thalamus [Montreal Neurological Institute (MNI) coordinates: x = 23, y = -9, z = 10]. The 37 chronic patients (Supplementary Figure 2A, right panel) showed maximal lesion overlap in similar regions, centered on the subcortical nuclei putamen and caudate, as well as anteromedial temporal lobe [MNI coordinates: x = 36, y = -2, z = 3].

To further quantify damage differences and similarities across the two populations, we applied the Lesion Quantification Toolkit[3] (see also Methods). We parcelled (i) the gray matter and subcortical nuclei according to functional areas defined by the Glasser Atlas (Supplementary Figure 2B;[1,2]), (ii) the white matter into its main longitudinal tracts as delineated from high-resolution diffusion data in the human connectome project[4], and (iii) the white matter into its parcel-to-parcel connectivity matrices for a fine-grained quantifaction of disconnections. Then, we calculated the average percentage damage for each Glasser atlas parcellation, longitudinal tract, and parcel-to-parcel disconnection. Qualitatively, the chronic and acute populations showed a similar distribution of parcel damage, tract-disconnection severity, and parcel-to-parcel disconnection (Supplementary Figure 1C-D). In addition, we computed the percentage of lesion volume within white matter, cortical gray matter, subcortical structures, as well as their possible combinations, and four major supra- and infra-tentorial arterial territories: anterior, middle, posterior cerebral arteries, vertebro-basilar, as well as their combinations. These distributions did not significantly differ between the two groups (Mann–Whitney U test, $U > 628$, $p > 0.2$). Overall, the chronic population showed a higher degree of parcel and disconnection damage, but, consistent with the voxel-based overlap analysis, their global anatomical distributions did not differ ($p > 0.05$, Bonferroni correction).

More generally, the voxel-based, parcel-based, disconnection-based patterns of lesions were very similar across the two populations, probably reflecting the general territorial organization of vasculature affected by naturally occurring stroke.

Supplementary Table 1. Neuropsychological tests used to quantify visual impairments.

Tests	Scores	Sample range	References
Line bisection (5 cm & 10 cm)	Rightward or leftward deviation from the true center (in millimeters)	5 cm: 0.0~8.0 mm<br>10 cm: 0.0~20.5 mm	Azouvi et al., 2002 & 2006
Apples cancellation	*Egocentric errors (CoC):* omissions of targets on the contra-lesional side of the sheet<br>*Allocentric errors (CoC):* incorrect selection of apples opened on the contra-lesioned side of the apples	-0.13~0.89<br>-0.04~0.83	Bickerton et al., 2011<br>Rorden and Karnath, 2010
Clock drawing	*Egocentric asymmetry*: left to right asymmetry of hours, omissions, or translocations<br>*Margin asymmetry*: distance between the right clock circumference and the rightmost hour – distance between the left clock circumference and the leftmost hour	-2.5 ~ 3.0<br>-0.1 ~ 0.5	Kaplan, 1990<br>Ronchi et al. 2019
Montreal cognitive assessment	*Cube copying*: one point for unsuccessful copying<br>*Clock drawing*: one point for the omission of per dimension-contour, numbers, and hands of clock	0 ~ 1<br>0 ~ 3	Nasreddine et al., 2005

Supplementary Table 1. Neuropsychological tests used to quantify visual impairments. *Tests* column indicates the names of neuropsychological tests used in the current study. *Scores* column indicates the scoring methods adopted for each test. *Sample range* column lists the range of scores from the minimum to the maximum scores in our stroke population. *References* column lists the original papers describing the adopted neuropsychological tests. CoC, Center of Cancellation.

A

Test overview

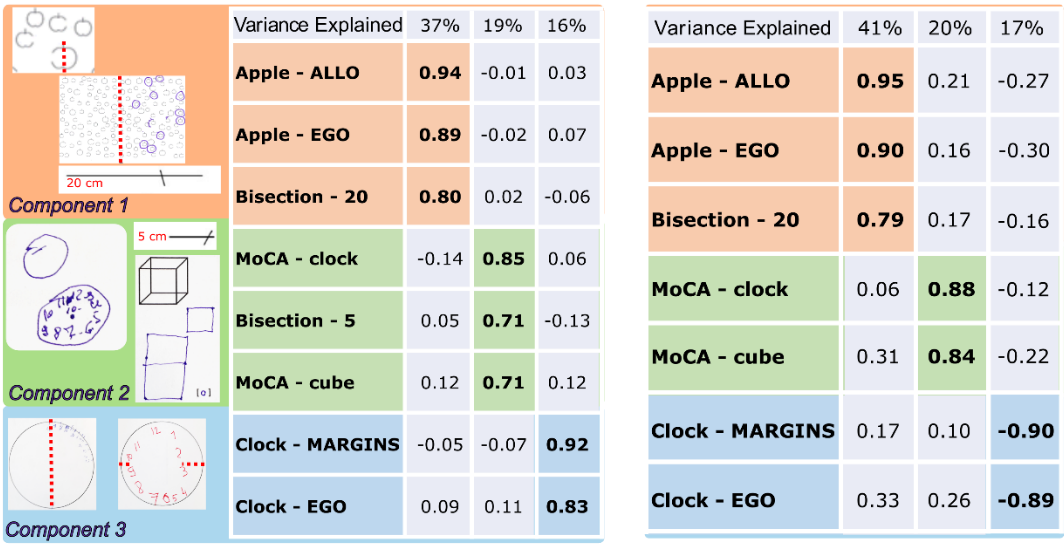

With bisection 5

Test name	PCA values		
Variance Explained	37%	19%	16%
**Apple - ALLO**	**0.94**	-0.01	0.03
**Apple - EGO**	**0.89**	-0.02	0.07
**Bisection - 20**	**0.80**	0.02	-0.06
**MoCA - clock**	-0.14	**0.85**	0.06
**Bisection - 5**	0.05	**0.71**	-0.13
**MoCA - cube**	0.12	**0.71**	0.12
**Clock - MARGINS**	-0.05	-0.07	**0.92**
**Clock - EGO**	0.09	0.11	**0.83**

Without bisection 5

Test name	PCA values		
Variance Explained	41%	20%	17%
**Apple - ALLO**	**0.95**	0.21	-0.27
**Apple - EGO**	**0.90**	0.16	-0.30
**Bisection - 20**	**0.79**	0.17	-0.16
**MoCA - clock**	0.06	**0.88**	-0.12
**MoCA - cube**	0.31	**0.84**	-0.22
**Clock - MARGINS**	0.17	0.10	**-0.90**
**Clock - EGO**	0.33	0.26	**-0.89**

B

Test overview

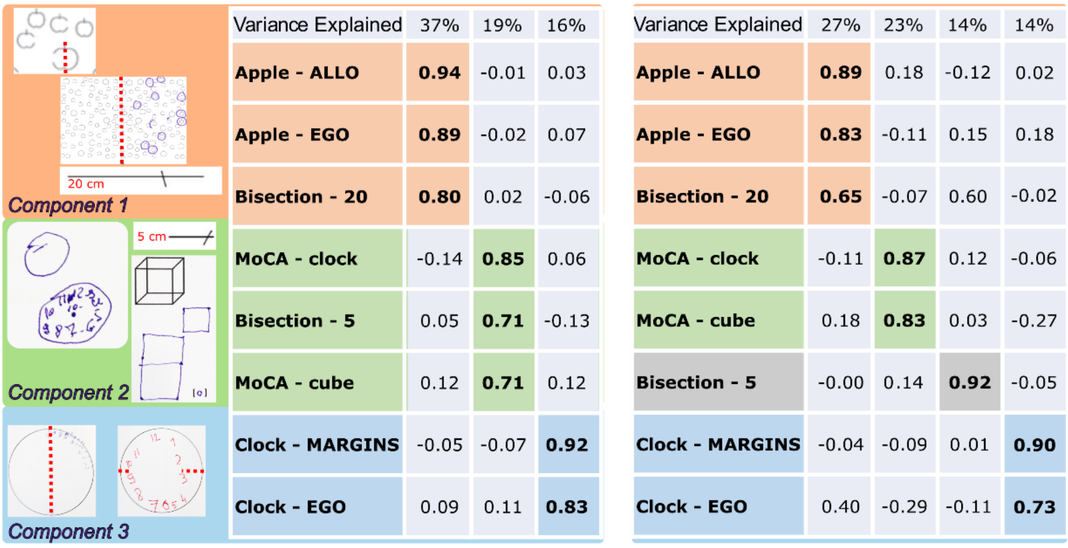

Whole population

Test name	PCA values		
Variance Explained	37%	19%	16%
**Apple - ALLO**	**0.94**	-0.01	0.03
**Apple - EGO**	**0.89**	-0.02	0.07
**Bisection - 20**	**0.80**	0.02	-0.06
**MoCA - clock**	-0.14	**0.85**	0.06
**Bisection - 5**	0.05	**0.71**	-0.13
**MoCA - cube**	0.12	**0.71**	0.12
**Clock - MARGINS**	-0.05	-0.07	**0.92**
**Clock - EGO**	0.09	0.11	**0.83**

Excluded patients with neglect deficits in MoCA

Test name	PCA values			
Variance Explained	27%	23%	14%	14%
**Apple - ALLO**	**0.89**	0.18	-0.12	0.02
**Apple - EGO**	**0.83**	-0.11	0.15	0.18
**Bisection - 20**	**0.65**	-0.07	0.60	-0.02
**MoCA - clock**	-0.11	**0.87**	0.12	-0.06
**MoCA - cube**	0.18	**0.83**	0.03	-0.27
**Bisection - 5**	-0.00	0.14	**0.92**	-0.05
**Clock - MARGINS**	-0.04	-0.09	0.01	**0.90**
**Clock - EGO**	0.40	-0.29	-0.11	**0.73**

Supplementary Figure 1: Control behavioral assessments of sample subpopulations (A) Control PCA (N=77 participants) testing the impact of excluding the short line bisection test — a measure with less normative data than the long line bisection test and known to be influenced by cross-over effects[5]. **(B)** Left panel: PCA based on the whole population (N = 77). Right panel: control PCA testing the impact of excluding a subpopulation of patients exhibiting signs of neglect deficits on the clock drawing and chair/cube drawing subtests from the MoCA battery (10 patients were excluded, resulting in N = 67 participants included in the control PCA shown in the right panel). In both panels, the tables display the principal components, variance explained, eigenvalues, and factor loadings obtained via factor analysis of various score types and subgroups. The first column presents an overview and individual examples of tests regrouped according to the main PCA analysis shown in the corresponding figure. No permutation testing was performed, as these analyses were conducted as control checks for the robustness of the main PCA results. PCA, principal components analysis; Apple, apple cancellation test; ALLO, allocentric; EGO, egocentric; Bisection-20: line bisection test in 20 centimeters length; MoCA-clock, Montreal Cognitive

Assessment-clock drawing test; Bisection-5: line bisection test in 5 centimeters length; MoCA-cube, Montreal Cognitive Assessment-cube copying test; clock, clock drawing test.

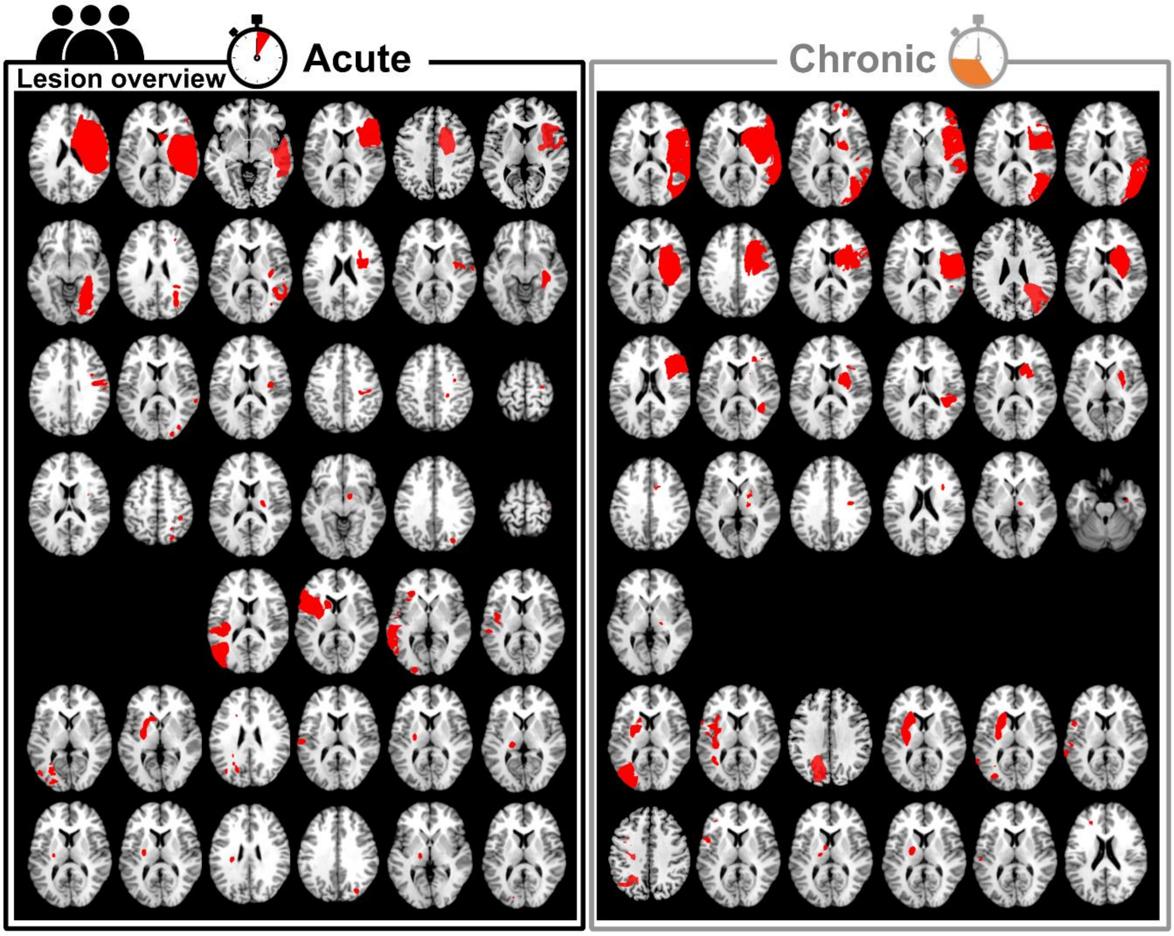

Supplementary Figure 2: Lesion Overview. Size and location of individual lesions for acute (left) and chronic (right) subgroups. Each slice depicts a single patient; right-lesioned patients and left-lesioned patients from each population are shown at the top and at the bottom of each panel, respectively.

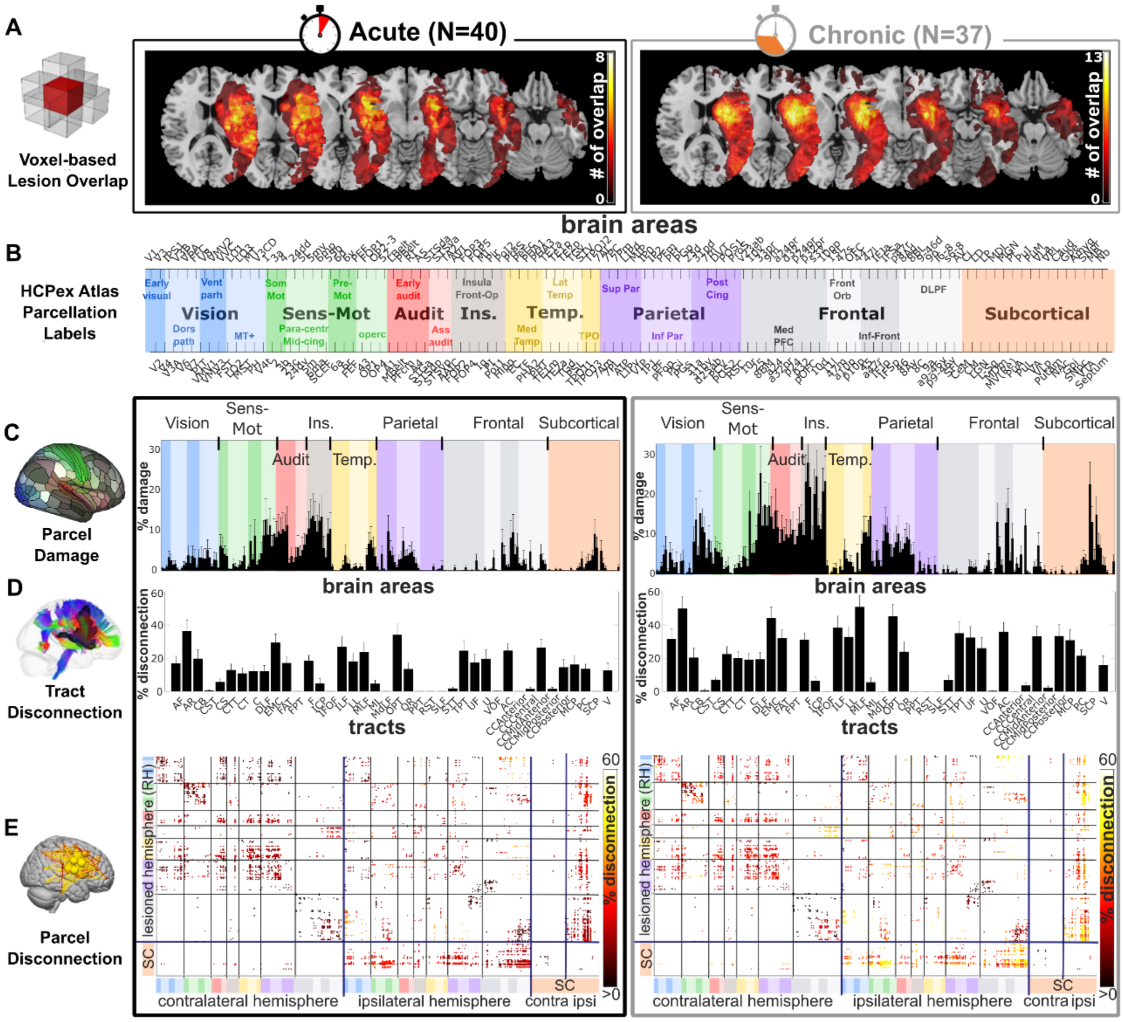

Supplementary Figure3: Lesion and disconnection patterns; The leftmost column provides the panel title and a schematic represantion of it for illustrative purposes only; the middle and right columns show data for the acute (N = 40) and chronic (N = 37) sub-populations, respectively. (A) **Volxel-based Lesion Overlap:** Colorbar indicates, for each voxel, the number of patients with a lesion at that location. (B) **HCPex Atlas Parcelation Labels**: Individual parcel names and the functional regrouping as provided by the HCPex atlas[1,2]. (C) **Parcel Damage**: Histograms report the percentage of damaged voxels in each Glasser parcel. (D) **Tract Disconnection:** Histograms report the percentage of disconnection across 72 major white-matter tracts. (E) **Parcel Disconnection:** Matrix plots show the percentage of disconnection for each parcel-to-parcel connection in the Glasser atlas; each colored cell marks a disconnected connection, and the colorbar encodes its severity. In total, 90,525 unique connections are shown (from 426 parcels). Statistics: Mann-Whitney U tests showed no significant difference in lesion volume size (U = 556.0, p > 0.05), parcel damage percentage (U > 561.5, p > 0.05), or tract-based disconnection percentage (U > 453.5, p > 0.05) between acute and chronic groups after Bonferroni correction for multiple comparisons. Sens-Mot, sensory-motor; Audit., auditory; Ins., insular; Temp., temporal; RH, right hemisphere; SC, subcortical; contra, contralateral; ipsi, ipsilateral. Parcels' abbreviations can be found here (link).

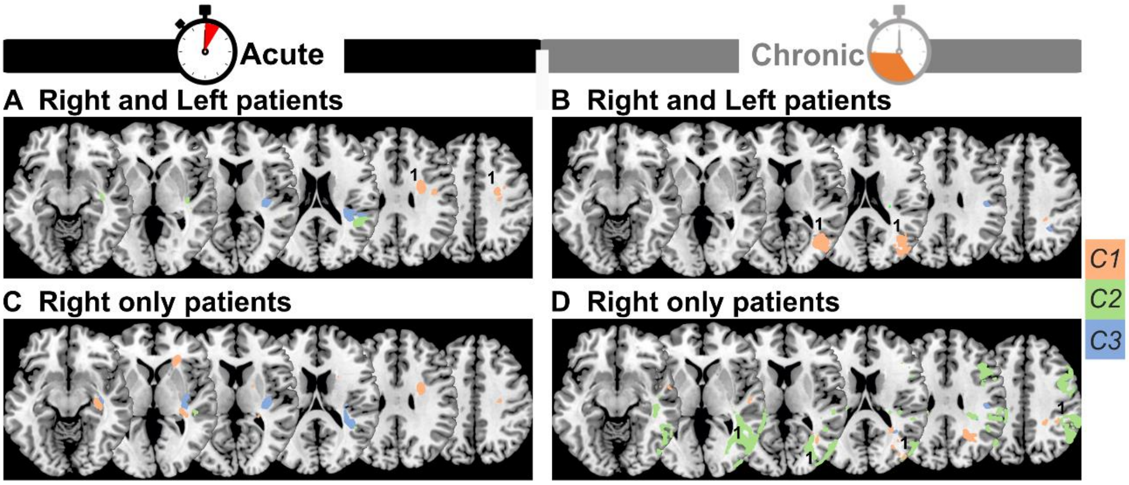

Supplementary Figure 4: Voxel-based lesion symptom mapping for right-only acute and chronic populations. Significant beta value results from the support vector regression model for the three behavioral components of deficits in the acute (A-C) and chronic (B-D) populations (only the voxels that survived the permutation-based family-wise error (FWE) correction ($p < 0.005$, one-tailed) are shown. Orange (C1), lateralized global/local inattention; green (C2): constructional deficit; blue (C3): lateralized within-object bias. All clusters survived after voxelwise correction ($p < 0.005$). Numbers indicate clusters that also survived after clusterwise correction ($p < 0.05$) based on 5000 permutations (see Methods). C1, component 1; C2, component 2; C3, component 3.

Supplementary Table 2. Major voxel and parcel damage from SVR-LSM lesion-symptom mapping.

	Acute				Chronic			
	voxel-based	right-only	marco-parcels	individual parcels	voxel-based	right-only	marco-parcels	individual parcels
GLOBAL/LOCAL INATTENTION	Precentral (#voxels 221)	Hippocampus (351)	Somatosensory/ Motor	1, 2, 3a, 3b, 4	Middle temporal (1395)	Superior parietal (978)	Temporo-Parieto-Occipital Junction	PSL
	Superior frontal (127)	Caudate (28)	Paracentral Lobular/Mid Cingulate	6mp	Middle occipital (923)	Superior temporal (451)	Dorsolateral Prefrontal	i6-8
	Middle frontal (82)		Premotor	55b, 6a, 6d, 6r, 6v, **FEF*, PEF***	Angular (75)	Middle occipital (206)	Primary Visual	V1
	Postcentral (54)		Posterior Opercular	43, FOP1, OP1, OP2-3, OP4	Inferior Parietal (68)	Precuneus (101)	Early Visual	V2, V3, V4
			Early Auditory	52, A1, LBelt, MBelt, PBelt, PFcm, RI			Ventral Visual Stream	V8
			Auditory Association	A4, STGa, TA2			MT+ / Neighboring Visual Areas	LO1, LO3
			Insular and Frontal Opercular	FOP2, Ig, PI, Pir, Pol1, Pol2			Auditory Association	STSda
			Inferior Parietal	PF, PFop, PFt			Medial Temporal	Hipp,
			Inferior Frontal	IFJp			Lateral Temporal	TE1m, TE1p, TE1a
			Dorsolateral Prefrontal	46, 8Av, a9-46v, i6-8			Temporo-Parieto-Occipital Junction	TPOJ3
			Thalamic Nuclei	VLp, VPL			Orbital and Polar Frontal	47s
			Subcortical Region	Putam, Caud, Gpe, Nb			Inferior Frontal	47l
							Subcortical Region	NAc, Amyg
CONSTRUCTIONAL	Superior temporal (490)	Superior temporal (21)	Premotor	6a	No voxels on gray matter	Middle-inf-sup temporal (5621)	Early Visual	V4
	Supramarginal (54)		Early Auditory	52, MBelt, PBelt		Sup-inf parietal (4484)	MT+ / Neighboring Visual Areas	LO1, LO3, MST, MT, PH
	Insula (50)		Auditory Association	A4, STGa, STSda, STSdp, TA2		Supramarginal (3025)	Auditory Association	STSva, STSvp
	Middle temporal (20)		Insular / Frontal Opercular	PI		Post and precentral (3204)	Lateral Temporal	PHT, TE1m, TE1p
			Temporo-Parieto-Occipital Junction	TPOJ1		Middle-inf occipital (1339)	Temporo-Parieto-Occipital Junction	TPOJ2, TPOJ3
			Dorsolateral Prefrontal	i6-8		Middle-inf frontal (1597)		
						Angular (882)		
						Calcarine (573)		
OBJECT-SPACE INATTENTION	Insula (312)	Insula (539)	Premotor	6a	Inferior parietal (135)	Post-central (164)	MT+ / Neighboring Visual Areas	LO1, LO3
	Superior temporal (140)	Putamen (192)	Early Auditory	52, LBelt, MBelt, PBelt	Superior parietal (119)		Superior Parietal	AIP
	Supramarginal (78)	Rolandic operculum (25)	Auditory Association	A4, STGa, TA2	Post-central (108)			
	Rolandi operculum (29)		Insular / Frontal Opercular	PI, Pol1,	Angular cortex (20)			
			Temporo-Parieto-Occipital Junction	PSL				
			Dorsolateral Prefrontal	i6-8				

Supplementary Table 2. Major voxel and parcel damage from SVR-LSM lesion-sim[ptom mapping. *Voxel-based* column indicates brain regions emerging from SVR-LSM after multi-comparison correction ($p < 0.005$, FWE corrected) based on 5000 permutations in all of the acute or chronic stages of our stroke patient sample. *Right-only* column lists the brain regions emerging from SVR-LSM after multi-comparison correction ($p < 0.005$, FWE corrected) based on 5000 permutations in the acute or chronic stroke patients with only right hemisphere brain lesions. *Macro-parcels* lists broad functional regions from the HCPex atlas, while *individual parcels* column lists fine-grained parcels within these categories. The parcels listed here from HCPex survived after 5000 permutation tests ($p < 0.05$, uncorrected). The colored rows indicate the three components regrouping different

visuospatial deficits; orange: lateralized global/local inattention; green: constructional component; blue: lateralized object-space component. Parcels' abbreviations can be found here (link).

Supplementary Table 3. Major tract- and parcel-to-parcel disconnection from SVR-LSM disconnection-symptom mapping.

	Acute		Chronic	
	tract-disconnection	parcel to parcel Disconnection	tract-disconnection	parcel to parcel Disconnection
GLOBAL/LOCAL INATTENTION	Fornix * (*p* = 0.001, corrected *p*= 0.027)	L_FEF to R_PEF (*p* = 0.0006)	Corticostriatal pathway (*p* = 0.013)	R_TPOJ2 to R_p47r (*p* = 0.0006)
	Corticothalamic pathway (*p* = 0.001)	R_a47r to R_Caud (*p* = 0.0008)	Optic Radiation (*p* = 0.019)	L_STSva to R_V3A (*p* = 0.0012)
	Corticostriatal pathway (*p* = 0.025)	L_FEF to R_55b (*p* = 0.0012)	Acoustic Radiation (*p* = 0.004)	L_PH to R_7AL (*p* = 0.0014)
	Cingulum (*p* = 0.007)	R_4 to L_FEF (*p* = 0.0016)	Arcuate Fasciculus (*p* = 0.037)	L_TPOJ1 to R_7AL (*p* = 0.0014)
	Arcuate Fasciculus (*p* = 0.022)	L_55b to R_PEF (*p* = 0.0016)	Vestibulocerebellar (*p* = 0.023)	R_LO2 to L_V3B (*p* = 0.0016)
	Frontal Aslant Tract (*p* = 0.023)			R_V2 to L_V6 (*p* = 0.0016)
	Posterior Commissure (*p* = 0.009)			
	Posterior Corpus Callosum (*p* = 0.043)			
	Anterior Commissure (*p* = 0.044)			
	Central Tegmental Tract (*p* = 0.002)			
	Inferior Cerebellar Peduncle (*p* = 0.041)			
CONSTRUCTIONAL	Inferior Cerebellar Peduncle (*p* = 0.032)	L_PGI to R_STSva (*p* = 0.0115)	Optic Radiation (*p* = 0.017)	R_V3B to R_FST (*p* = 0.0087)
		R_PGi to R_PuL (*p* = 0.0118)	Vestibulocerebellar (*p* = 0.020)	R_FST to R_V3CD (*p* = 0.0087)
		L_V3A to R_STSva (*p* = 0.0119)		R_V3CD to R_PHT (*p* = 0.0087)
		L_STSva to R_STSva (*p* = 0.0119)		R_PH to R_V3B (*p* = 0.0009)
		L_PF to R_TE1p (*p* = 0.0161)		R_MST to R_V7 (*p* = 0.0009)
				R_V3CD to R_LO3 (*p* = 0.0009)
OBJECT-SPACE INATTENTION	No tracts survived	R_PGI to R_PuL (*p* = 0.0104)	Optic Radiation (*p* = 0.030)	L_V3B to R_V1 (*p* = 0.0171)
		L_PGI to R_STSva (*p* = 0.0149)	Vestibulocerebellar (*p* = 0.030)	L_V7 to R_V1 (*p* = 0.0171)
		L_PF to R_TE1p (*p* = 0.0152)		R_V7 to R_FST (*p* = 0.0203)
		L_V3A to R_STSva (*p* = 0.0153)		L_V4 to R_V7 (*p* = 0.0215)
		L_STSva to R_STSva (*p* = 0.0116)		R_V3B to R_MST (*p* = 0.0229)

Supplementary Table 3. Major tract- and parcel-to-parcel disconnection from SVR-LSM disconnection-symptom mapping. *Tract-disconnection* column represents tracts survived in SVR-LSM after 5000 permutation tests ($p < 0.05$, uncorrected). *Parcel-to-parcel disconnection* column lists the top 5 severe disconnections in paired parcels from HCPex atlas, surviving after 5000 permutation tests ($p < 0.05$, uncorrected). The colored rows indicate the three components regrouping different visuospatial deficits; orange: lateralized global/local inattention; green: constructional component; blue: lateralized object-space inattention. * means survived after multi-comparison correction ($p < 0.05$, FWE corrected) based on 5000 permutations. Parcels' abbreviations can be found here (link).

Supplementary References

1. Glasser MF, Coalson TS, Robinson EC*, et al*. A multi-modal parcellation of human cerebral cortex. *Nature*. 2016;536:171-178.
2. Huang CC, Rolls ET, Feng J, Lin CP. An extended Human Connectome Project multimodal parcellation atlas of the human cortex and subcortical areas. *Brain Struct Funct*. 2022;227:763-778.
3. Griffis JC, Metcalf NV, Corbetta M, Shulman GL. Lesion Quantification Toolkit: A MATLAB software tool for estimating grey matter damage and white matter disconnections in patients with focal brain lesions. *NeuroImage: Clinical*. 2021;30:102639.
4. Yeh, Panesar S, Fernandes D*, et al*. Population-averaged atlas of the macroscale human structural connectome and its network topology. *Neuroimage*. 2018;178:57-68.
5. Doricchi F, Guariglia P, Figliozzi F, Silvetti M, Bruno G, Gasparini M. Causes of cross-over in unilateral neglect: between-group comparisons, within-patient dissociations and eye movements. *Brain*. 2005;128:1386-1406.
